# Concerning the Liver

**Published:** 1871-02

**Authors:** 


### CONCERNING THE LIVER.

After food is eaten, it is made into blood, and sent direct to the liver to be filtered; the office of the liver being to withdraw from the blood all that is waste, impure, and imperfect; this filtered blood is then sent to the lungs through the heart, to be prepared still more perfectly for imparting health, and strength, and warmth, and life to the whole body. Hence, if the liver does not do its work, it is said not to "act," to be "torpid," to be "lazy," to be "asleep;" and the man soon becomes "bilious," the blood becomes so impure, so heavy, so thick, that the whole body becomes heavy, the head aches, the mind is depressed, there is no life, no animation, no appetite. This waste and impure matter which is withdrawn from the blood is called the "bile," is collected together in a little receptacle holding a few table-spoons, called the

### GALL BLADDER.

The almost universal "symptom" or sign that the liver is not doing its work properly, is an aversion to food, accompanied with a kind of sickish feeling; the very thought of eating is almost enough to cause vomiting. The point in all such cases is first to carry out Nature's instinct by eating nothing whatever until the appetite becomes voracious, keeping abundantly warm all the time. If persons are in a hurry to get well, then exercise in the open air from sunrise to sunset; exercise moderately, steadily, in something which is agreeable, and, better still, profitable.

The advantage of this method of causing the liver to work is that each additional use of the remedy, if promptly applied the very hour the liver is noticed to be "out of order," will make the cure surer, easier, quicker, safer, every time.

Exercise makes the liver "work," because in exercise all the muscles of the body are put in motion, and this motion is communicated to the machinery within. The exercise should be out of doors, because out-door air is purest, goes pure into the lungs, but comes out so impure that if re-breathed without any admixture of pure air, we would "faint" on the spot. The air going out being so freighted with the impurities of the blood, it in a sense lightens the burden of the liver, helps to unclog its machinery, and it starts off to work itself with almost the instinct that an animal rises from the earth when pressed down to it with an over-weight, the moment that weight is lightened. But there is a still more speedy method of making the liver "work."

This important organ, the largest manufactory of the body, is at the lower edge of the ribs on the right side, extending nearly from the right hip over towards and near the navel. In one sense it is like a sponge, which, if pressed upon, parts with the liquid which it contains; or like a bladder with an open mouth, pressure "squeezes" out its contents. Then with the ball of the hand, at the body end of the thumb, press in towards the liver and downward, beginning at the right hip and coming round to the centre of the body over the stomach; press firmly, aiding the pressure with the other hand if you choose; do this eight or ten minutes night and morning; this may be called "kneading the liver," and is an excellent substitute for medicine. To persons who are troubled a great deal with biliousness, this is a very efficient means of remedying the trouble.

A more speedy method still of making the liver work is to take medicines which are known to "act" on the liver, but this becomes more properly the province of the physician to advise, with this warning to all who prefer taking medicine: each successive dose must be larger, in order to accomplish the same object, while the intervals become shorter, until after a while the man is all the time taking medicine, and is all the time sick; hence the unmedicinal means of keeping the liver at work are safest and best, because they offer no violence to the system, and become more and more efficient at each repetition. But sometimes life can be saved only by giving relief within a few minutes; this is done by giving an

EMETIC, the operation of which is to give a heave; this brings the stomach and other muscles up and against the liver, making a kind of pressure, as with the ball of the hand mentioned a while ago, but more efficiently; in fact, so much so, that the gall bladder sends its contents toward the stomach, causing by its presence there such deathly sickness that out they come through the mouth as "bitter as gall," literally. But as this heaving and straining has sometimes ruptured a blood-vessel, causing a bleeding to death, the reader is advised never to take an emetic on his own responsibility to make the liver work promptly, but rather consult a physician, or use the milder means previously named.

